# Phenolic composition, antioxidant, anti-wrinkles and tyrosinase inhibitory activities of cocoa pod extract

**DOI:** 10.1186/1472-6882-14-381

**Published:** 2014-10-07

**Authors:** Azila Abdul Karim, Azrina Azlan, Amin Ismail, Puziah Hashim, Siti Salwa Abd Gani, Badrul Hisyam Zainudin, Nur Azilah Abdullah

**Affiliations:** Cocoa Innovation and Technology Centre, Malaysian Cocoa Board, PT12621, Nilai Industrial Area, 71800 Nilai, Negeri Sembilan Malaysia; Department of Nutrition and Dietetics, Faculty of Medicine and Health Sciences, Universiti Putra Malaysia, 43400 UPM Serdang, Selangor Malaysia; Laboratory of Halal Science Research, Halal Products Research Institute, Universiti Putra Malaysia, 43400 UPM Serdang, Selangor Malaysia; Research Centre of Excellence, Nutrition and Non-communicable Disease, Faculty of Medicine and Health Sciences, Universiti Putra Malaysia, 43400 UPM Serdang, Selangor Malaysia

**Keywords:** Cocoa pod extract, Antioxidant, Anti-wrinkles, Skin whitening, Sunscreen agent, Cosmetic ingredient

## Abstract

**Background:**

Cocoa pod is an outer part of cocoa fruits being discarded during cocoa bean processing. Authors found out that data on its usage in literature as cosmetic materials was not recorded in vast. In this study, cocoa pod extract was investigated for its potential as a cosmetic ingredient.

**Methods:**

Cocoa pod extract (CPE) composition was accomplished using UHPLC. The antioxidant capacity were measured using scavenging assay of 1,2-diphenyl-2-picrylhydrazyl (DPPH), β-carotene bleaching assay (BCB) and ferric reducing antioxidant power (FRAP). Inhibiting effect on skin degradation enzymes was carried out using elastase and collagenase assays. The skin whitening effect of CPE was determined based on mushroom tyrosinase assay and sun screening effect (UV-absorbance at 200-400 nm wavelength).

**Results:**

LC-MS/MS data showed the presence of carboxylic acid, phenolic acid, fatty acid, flavonoids (flavonol and flavones), stilbenoids and terpenoids in CPE. Results for antioxidant activity exhibited that CPE possessed good antioxidant activity, based on the mechanism of the assays compared with ascorbic acid (AA) and standardized pine bark extract (PBE); DPPH: AA > CPE > PBE; FRAP: PBE > CPE > AA; and BCB: BHT > CPE > PBE. Cocoa pod extract showed better action against elastase and collagenase enzymes in comparison with PBE and AA. Higher inhibition towards tyrosinase enzyme was exhibited by CPE than kojic acid and AA, although lower than PBE. CPE induced proliferation when tested on human fibroblast cell at low concentration. CPE also exhibited a potential as UVB sunscreen despite its low performance as a UVA sunscreen agent.

**Conclusions:**

Therefore, the CPE has high potential as a cosmetic ingredient due to its anti-wrinkle, skin whitening, and sunscreen effects.

## Background

Skin wrinkles form as a result of natural aging process and the presence of excessive amount of reactive oxygen species (ROS) [1]. Although skin has self-defence system to deal with ROS, excessive and chronic exposure to UV can overwhelm the condition leading to oxidative stress and damage resulting premature aging. An imbalance of ROS on skin is due to factors such as overexposure to sunlight [2] and lack of essential nutrient intake [3]. In normal condition, skin produces enzymes such elastase and collagenase, at similar rate as aging process occurs and age increases. However, with overexposure to sunlight (UVA and UVB), the presence of excessive ROS and smoking habit [4], the enzymes are produced at a faster rate resulting in faster degradation of elastin and collagen which are the main foundation of extracellular matrix (ECM) of the dermis [5]. Additionally, excessive exposure to sunlight, induced production of melanin in the skin layer. Tyrosinase is the responsible enzyme that initiates skin pigmentation [6]. Potentially, plant extracts can suspend the skin aging and pigmentation process [7] by encountering the ROS [3] and protecting the skin from UV [1]. The extracts protect the skin in various ways, including scavenging the ROS, reducing the ROS reactivity, inhibiting the oxidation, absorbing the UV light, suppressing the enzymes, thus reducing the risk of wrinkle formation and protecting the skin from aging. Examples of compounds that have attracted researchers to topical skin applications are polyphenols, triterpenes and stillbenoids. Polyphenols have a phenol ring with at least one hydroxyl substituent that enables scavenging of ROS, reducing metal ions, modulating protein phosphorylation (related to inhibition of enzymes activity), and inhibiting lipid peroxidation [8].

Cocoa pods are agricultural waste abundantly produced in cocoa plantations during the extraction of cocoa beans for cocoa processing. As discarded materials, cocoa pods can initiate the black pod disease by making the ground susceptible to the growth of fungi, which is typical in hot and humid climate. The pods vary in color (from maroon to green) and thickness when ripe depending on their clone. In Malaysia, multi clones of cocoa are planted and when discarded, the pods are thrown away in bulk regardless of the clones. Cocoa pods are used as fertilizers and animal feed. It is also being used as a source of activated carbon (unpublished data), potash, colorant [9], gum [10] and anti hypercholesterolemia supplement [11]. Previously, total phenolic content of cocoa pod husk was determined at 45.6-46.4 mg gallic acid equivalent of soluble phenolic while 32.3% carbohydrate, 21.44% lignin, 19.2% sugars, 8.6% protein and 27.7% minerals were reported [12]. Higher level of antioxidant in cocoa pod extract by total phenolic content (TPC) was reported at 49.54 ± 3.39 mg gallic acid equivalent (GAE) per gram sample and total flavonoid content (TFC) at 22.42 + 0.98 mg rutin equivalent per gram sample (unpublished data). Sartini et al. [11] also reported higher TPC value of cocoa pod husk at 56.5 ± 0.57 mg GAE/g. Scientific publication on identification of polyphenol compounds in a cocoa pod is rare, necessitating further research. Therefore, the present experiment was carried out to determine the cocoa pods compounds, which are rich in antioxidant activity and can be turned into high end-value products. In this paper, we investigated the anti-wrinkle, skin whitening and potential UV-protecting properties of cocoa pod extract using in-vitro assays and identified the potential of this extract as a new active ingredient for cosmetic.

## Methods

### Reagents

Reagents used were of analytical grade purchased from suppliers such as Sigma-Aldrich for 1,2-diphenyl-2-picrylhydrazyl (DPPH), L-(+)-ascorbic acid (AA), Linoleic acid, Kojic acid (KA), Tyrosinase (from mushroom) and L-3,4-dihydroxyphenyl-alanine methyl ester hydrochloride (L-DOPA); Merck for acetonitrile, ammonium formate, formic acid, dimethylsulfoxide (DMSO), hydrochloric acid (HCl), chloroform and Tween-20; Fluka for 2,4,6-tripyridyl-s-triazine (TPTZ); Qrec for ferric chloride (FeCl_3_), Friendemann Schmidt for ferrous sulfate (FeSO_4_.7H_2_O), GmbH for ethanol and methanol; and MP Biomedicals for β-carotene powder. Test kits purchased from Enzo Life Sciences, Inc. Standardized Pine Bark extract (PBE) sample was provided by DKSH Malaysia Sdn Bhd (sample from Horphag, Switzerland) and used as a positive control in addition to ascorbic acid, and butylated hydroxytoluene (BHT, Sigma-Aldrich) in β-carotene bleaching assay. Human dermal fibroblast, adult (HDFa) was acquired from Life Technologies Corporation (GIBCO, catalogue number C-013-5C; Lot number 1378119) as well as Dulbecco’s modified Eagle’s medium (DMEM) and Trypsin (Trypsin LE™). Fetal bovine serum (FBS) and Streptomycin/Penicillin (antibiotics) obtained from Biowest. MTT or 3-(4,5-Dimethylthiazol-2-yl)-2,5-diphenyl tetrazolium bromide supplied by Bio Basic Canada Inc. Phosphate Buffer Saline (PBS, 10X) purchased from R&M Chemicals, UK.

### Sample preparation

Discarded cocoa pods collected from a cocoa fermentation site at cocoa plantation in Cocoa Development and Research Centre, Jengka, Pahang, Malaysia. After rinsed with tap water, the pods were chopped using mechanical fruit slicer (FC-312, Zhaoqing Fengxiang Food Machinery, China) for better water removal when drying in high-performance dryer (FD-825, Protech, Malaysia). The dried pods were ground into powder at 1.0 mm size using a grinding machine with a sieve (Automatic Hammer Mill Grinder, China). The cocoa pod powder was kept in tight containers at room temperature (not more than two weeks) until extraction procedure adjourned.

Twenty millilitres of aqueous ethanol (80%) were poured into a conical flask containing one gram of cocoa pod powder and shaken in water bath shaker (BS-21, Lab Companion, Korea) at 120 rpm, 40°C, for 30 minutes to prepare the extract. Soluble portion filtered (using Whattman No.1), evaporated to dryness (using vacuum rotary evaporator; IKA, Germany) and re-dissolved with 5 ml ethanol after recording the weight. Insoluble material was filtered out using filter paper. The extracted sample was stored in air-tight vial at -10°C until evaluated. We used AA and PBE as the positive control in the study, due to its effective role in maintaining cellular function [13, 14] which affects the skin condition.

### Identification of CPE compound using LC/MS/MS

LC-MS/MS analysis was performed using a Perkin Elmer Flexar FX-15 ultra-high performance liquid chromatography (Perkin Elmer, USA). It equipped with a reversed-phase C18 analytical column of 50 mm × 2.1 mm × 1.9 μm particle size (Perkin Elmer, USA). The column oven temperature was set at 35°C, and the flow rate was 250 μl/min. Mobile phases A and B were water and acetonitrile, respectively, each containing 5 mM ammonium formate and 0.1% formic acid. The linear gradient programme was set as follows: 0–0.1 min, 90% A/10% B; 0.1-8 min, 10% A/90% B; 8–10 min, 10% A/90% B; 10–15 min, 90% A/10% B. The injection volume was 20 μL with a run time of 15 minutes. The UHPLC was hyphenated to a triple quadrupole mass spectrometer 3200 QTrap (ABSciex) equipped with an electrospray ionization interface set at negative mode. The interface heater held at the temperature of 500°C and an ion-spray (IS) voltage of -4500 eV. The nebulising gas (GS1), heating gas (GS2) and curtain gas pressures set at 40, 40 and 10 psi, respectively during the whole analysis. Nitrogen was used as collision and spray gas. Full scan data acquisition was performed, scanning from m/z 5 to 1500 in enhanced MS IDA EPI mode. Analyst software version 1.5.2 was used for method development, data acquisition and data processing.

### Determination of antioxidant activities

Antioxidant activities were evaluated using three different assays; i.e., 1,2-diphenyl-2-picrylhydrazyl (DPPH), Ferric Reducing Antioxidant Power (FRAP) and β-carotene bleaching assay (BCB). Methods of preparation of DPPH [2, 15, 16], FRAP [17–22] and BCB [23] solutions and calculation were adopted with some modifications. Briefly, DPPH solution was prepared by diluting 1.2 ml of DPPH stock solution (0.2 M DPPH in ethanol) with 3 ml ethanol and 0.5 ml DMSO. In a 96-well microplate, 270 μl of the solution was added to 30 μl of tested sample in a dilution series of 7.8-1000 μg/ml, and absorbance measured at 550 nm [24]. Scavenging effect was calculated by the percentage of faded purple DPPH solution color into yellow by the tested sample against the control (DPPH solution only). The EC_50_ of DPPH assay represents the concentration of the tested sample needed to reduce the DPPH by 50% where the value obtained from linear regression graph.

For FRAP assay, two solutions; i.e., ferric chloride solution (3 mM in 5 mM citric acid) and TPTZ solution (2,4,6-tripyridyl-s-triazine; 1 mM in 0.05 M hydrochloric acid) were prepared. Fifteen μl of the tested sample was added to 270 μl TPTZ solution and measurement at 620 nm was carried out immediately after addition of 15 μl of ferric chloride solution. The absorbance data calculated against serial dilution of Ferrous sulfate (FeSO_4_.7H_2_O) and recorded as equivalent to μM Fe^2+^. Effective concentration at 50% (EC_50_) of FRAP value is the sample concentration required to reduce 0.5 mol of Fe^3+^ to Fe^2+^.

Two milligrams of β-carotene powder was dissolved in 0.2 ml chloroform followed by adding 0.2 ml linoleic acid, 2 ml Tween20 and 100 ml of distilled water to make β-carotene solution. It was mixed until almost transparent solution obtained. Two hundred μl of the solution was added to 20 μl of the tested solution and measurement at 450 nm was carried out after incubation for 20 minutes at 50°C. Measurement was monitored for 2 hours at 30-minute intervals. Calculation of antioxidant activity percentage obtained by the difference of degradation rate of tested sample to the degradation rate of control (β-carotene solution only). Effective concentration at 50% (EC_50_) was determined to represent the ability of the sample to protect the β-carotene solution from degradation, thus indicating high antioxidant activity with low concentration of the tested sample. Instead of AA, BHT was used as a positive control in this assay. BHT is well-known antioxidant specifically in protecting lipid oxidation [25] and stronger antioxidant compound compared with AA [26].

### Determination of elastase and collagenase inhibition

Elastase and collagenase inhibition measurements were carried out using drug discovery kits (Neutrophil Elastase Colorimetric and MMP-1 Colorimetric, respectively) following protocols as in Enzo Life Science [27, 28]. For elastase inhibition assay, 20 μl of tested sample was diluted with 65 μl buffer-solution containing 100 mM HEPES, 500 mM NaCl and 0.05% Tween20 in DMSO in a 96-well plate. Elastatinal (100 μM) used as the control inhibitor. The neutrophil elastase enzyme (purified human neutrophil elastase, 2.2 μU/μl) at 10 μl was added to the diluted tested sample and incubated for 10 minutes at 37°C. Later, 5 μl substrate (MeOSuc-Ala-Ala-Pro-Val-pNA, 100 μM) was added to each well and absorbance at 405 nm was monitored for 10 minutes.

Briefly for collagenase inhibition assay, 20 μl of tested sample was diluted with 50 μl buffer-solution (50 mM HEPES, 10 mM CaCl_2_, 0.05% Brij-35 and 1 mM DTNB in DMSO). Twenty microlitres of MMP-1 enzyme (*E.coli* recombinant human MMP-1 catalytic domain, 153 mU/μl) was added to each well prior to incubation at 37°C for 30 minutes. Control inhibitor, NNGH (N-Isobutyl-N-(4-methoxyphenylsulfonyl) glycylhydroxamic acid; 1.3 μM), was used for comparison. Substrate (thiopeptide, Ac-PLG-[2-mercapto-4-methyl-pentanoyl]-LG-OC_2_H_5_; 100 μM) at 10 μl was added to each well and absorbance at 410 nm was monitored for 10 minutes.

For both protocols, slope of remaining activity for the tested sample against the control (without sample) was calculated in percentage and inhibition percentage was obtained by subtracting the obtained value from 100. Inhibition concentration of 50% (IC_50_) is the concentration of the tested sample that can inhibit the enzymes activities to 50%.

### Determination of skin whitening and potential UV-sunscreen activities

Skin whitening effect was evaluated based on inhibition of mushroom tyrosinase by the tested sample with L-DOPA as substrate using a method described by Chiari et al. [29]. The tested solution was diluted in series (1000-250 μg/ml) using DMSO and 20 μl was pipetted into a 96-well microplate, followed by addition of 138 μl PBS (phosphate buffer solution) and 2 μl mushroom tyrosine solution (2500 U/ml, in PBS). After incubation at 37°C for 90 minutes, 40 μl of L-DOPA (2.5 mM in PBS) was added, and measurement at 450 nm monitored for 20 minutes. Kojic acid was used for comparison.

For UV sunscreen potential activity, the tested sample was dissolved in ethanol (ratio 8:125). Same solvent was used as blank for baseline correction so that the results obtained could be compared with each other. Samples were scanned at 200-400 nm wavelength using dual beam UV-Spectrophotometer (Cary 60, US). Absorbance of tested samples at critical wavelengths (290, 308, 330 and 350 nm) [30] were selected for comparison. Higher value of absorbance indicates better potential as UV-sunscreen agent, in-vitro. Two broad spectrums of commercial sunscreens; i.e. Avobenzone and Octylmethoxycinnamate (OMC) were used for comparison. The results obtained were only for screening purposes; therefore, a subsequent experimental study using non-invasive method had to be carried out to determine the Sun Protecting Factor (SPF) value prior to using the extracts in formulation.

### Cell viability using human dermal fibroblast

Healthy cells were initiated from cryopreserved HDFa in a 25 cm^2^ tissue culture flask in DMEM containing 10% FBS and 1% antibiotics. The cells were incubated at 37°C, 95% humidity and 5% CO_2_ until confluent. The growth media was refreshed every two days until at least 80% confluence was achieved, and was trypsinized with Trypsin LE™ to passage for not more than eight times for cell viability study [31]. The cells were seeded into a 96-well microplate at density of 1×10^5^ per well and incubated for 24 hours. Serial dilution of CPE, AA and PBE were added to the well, respectively, after removal of the spent media and incubated for another 24 hour. Forty microliters (40 μL) of MTT in PBS (2.5 mg/mL) was added to each well and incubated for 4 hours before the absorbance was measured at 570 with 630 nm as reference wavelength. A hundred microlitres (100 μL) of DMSO was used to dissolve the dye crystals [32]. The percentage of cell viability was calculated based on the optical density of each well against the control (cell without any treatment). Inhibition concentration of 90% (IC_90_) is the sample concentration that enables 90% of cells survived after treatment with the tested sample which metabolized the MTT salts to formazan [33].

### Statistical analysis

The results were presented as mean ± standard deviation determined in triplicates of two independent samples. Comparison was made using two samples *T*-test by Minitab Software version 14.12.0 (US Inc.). Results were significantly different when p-value was less than 0.05 (p < 0.05).

## Results and discussion

### Identification of CPE compound using LC/MS/MS

Crude CPE was analyzed by LC/MS/MS in negative mode. We identified each compound based on the literature search in established spectrum databases [34, 35]. Product ion spectrum of CPE was compared with the product ion spectrum obtained from the search and was listed in Table 1 [34–54] according to its retention time. We considered the compound as its derivatives, if the product ion spectrum did not hit any of the product ion m/z in the search but hit only the first and one or more of the product ion m/z in the spectrum. PBE, known to contain a wide range of polyphenol compounds [36], was also analyzed for comparison purposes. Compounds that are similarly available in PBE also were noted at the end column of Table 1. Additionally, compounds that appear only in PBE were listed in Table 2 [34, 35, 55–57]. CPE and PBE contained almost similar compounds, including polyphenols, carboxylic acid (citric acid and malic acid) and sugars (gluconic acid sodium salt). Polyphenolic compounds in CPE included phenolic acids (protocatechuic acid, p-hydroxybenzoic acid and salicyclic acid), flavonols (kaempferol) and flavones (linarin), which also were detected in PBE. Resveratrol, a stilbenoids compound that was detected in wine [37], was also found in CPE but not in PBE. CPE also was detected with tartaric acid, apigenin and luteoin (flavone), crysoplenol (terpenoids), linoleic acid and oleic acid (fatty acids) which were not available in PBE. Nevertheless, gallic acid, catechin [58], quercetin and caffeic acid were found in PBE but were absent in CPE.

**Table 1:** **Product ion of cocoa pod extract in negative mode**

***Class***	***Compound***	***Retention time, t***_***R***_ ***(min)***	***m/z***	***Reference***	***in PBE***
Sugar	Gluconic acid sodium salt/glucose acid	0.787	195,177,129,85,75	34,35	Yes
Carboxylic acid	Tartaric acid	1.184	149,105,87,73	34	No
Flavonoid/flavonol	Rhamnetin	1.315	315,241,139,108,97,80,70	34	No
Carboxylic acid	Citric acid	1.447	191,111,87	53,35	Yes
Phenolic acid	Protocatechuic acid	1.842	153,109,65	38,39	Yes
Phenolic acid	Protocathecuic derivatives/nucleotide	1.972	329,241,199,108,97,69	41/35	No
Phenolic acid	Protocathecuic derivatives/nucleotide	1.974	329,241,139,97,80	41/35	No
Unknown		2.106	330,241,122,96,80	-	No
Phenolic acid	p-hydroxybenzoic acid	2.238	137,93	36,40	Yes
Phenolic acid	Salicyclic acid	2.238	137,108,91,65	35	Yes
Unknown		2.500	363,241,122,96,59	-	No
Phenolic acid	Methyl salicylate	2.633	151,139,124,91,65	35	No
Nucleotide	disodium salt, nucleotide	2.763	401,327,69	35	No
Phospholipid	Phospholipid derivatives	2.897	381,249,161,113,98,85,68	35	No
Glucosinolate	Glucosinolate derivatives	3.029	379,155,59	35	No
Flavonoid	Sineginhomoorientin derivatives	3.161	355,225,207,96,69,59	35,42	No
Unknown		3.292	358,245,222,178,161,135,123	-	No
Unknown		3.293	358,222,178,161,151,135,123	-	Yes
Unknown		3.423	452,408,372,328,285,250,230,190,178,160,148,135	-	No
Flavones-Sugar	Apigenin-c-glucose-c-pentoside	3.425	563,545,473,443,395,383,365,353,325,311,297,233	43	No
Unknown		3.557	357,241,139,96	-	No
Glucosinolate	Glucosinolate derivatives	3.688	372.2,285.1,136,178,147,160.2,135	35	No
Glucosinolate	Glucosinolate derivatives	3.822	439.2,314,300.1,269.1,180.1,151.1,96.9,80.1	35,42	No
Glucosinolate	Glucosinolate derivatives	4.086	371,281,241,151,96	35	No
Terpenoid	Terpenoid derivatives	4.351	345,201,171,155,59	35	No
Flavonoid/Flavonol	Kaempferol derivatives	4.879	723,677,659,550,451,367,225	34	Yes
Terpenoid	Terpenoid derivatives	5.010	345,99,59	35	No
Flavonoid	Flavone derivatives	5.143	327,309,197	35	No
Unknown		5.404	329,229,211,171,139,99	-	No
Flavone	Flavone/luteolin	5.407	329,311,229,211,171,139,127,99,69	44	No
Flavonoid	Flavone derivatives	5.535	327,205,183,171,69,59	35	No
Ketone-Sugar	Ribulose1,5-bisphosphate	5.667	309,265,209,193,151,137,109	53	No
Terpenoid	Crysoplenol	5.802	330,172	35	No
Flavonoid	Flavones derivatives	6.199	305,287,249,163,135,93	37,54,45,46	No
Flavones-Sugar	apigenin glycoside	6.594	313,295,183,171,129,99,58	34,44	No
Phenylpropanoid	Chlorogenic acid derivatives	6.858	353,97,80	34	No
Unknown		7.377	339,239,183,170,99	-	Yes
Flavonoid	Flavones derivatives	7.384	295,277,181,171	35	No
Flavonoid	Flavones derivatives	7.516	293,249,195,113	35	No
Flavonoid	Flavones derivatives	7.516	293,249,236,220,205,190,177,164,148,81	35	No
Carboxylic acid	Malic acid	8.042	133, 115,71	34,47,48,49,53,35	Yes
Stillbenoids	Resveratrol	8.567	277	50	No
Stillbenoids	Resveratrol	8.567	227	37,51	No
Fatty acid	Linoleic acid	9.094	279	41	No
Vitamin	Flavin mononucleotide	9.218	455	43	No
Fatty acid	Oleic acid	9.620	281	41	No
Flavonoid/flavone	Linarin/acacetin	14.350	283	52	Yes

**Table 2:** **Extra compound of pine bark in negative mode**

***Class***	***Compound***	***Retention time, t***_***R***_ ***(min)***	***m/z***	***Reference***
Unknown		1.314	399,353,221,207,161,85	-
Phenolic acid	Gallic acid	1.446	169,125,95,79,67	35
Phenolic acid	Methyl benzoate acid/ caffeic acid	1.841	135, 108,93,80	34,55,56,57
Phenolic acid	3,5-dihydroxybenzoic acid	1.841	109,91,65	35
Unknown		2.369	445,137	-
Unknown		2.634	459,137,93	-
Unknown		3.564	466,438,303,285,275,231,175,151,125,82,57	-
Flavonoid/flavanol	Quercetin derivatives	4.229	506,459,340,151,165,125	35
Unknown		4.494	350,290,244,135	-
Unknown		5.159	291,247,217	-
Unknown		5.424	365,321,247,227,165,151	-
Flavonoid/flavanol	Catechin	5.424	289,173,162,137,122,109	35
Unknown		5.557	349,305,287,269,207,189,177,161,147,85	-
Unknown		5.689	329,255,227	-
Carboxylic acid	Gibbrellin derivatives	5.821	331,287,269,253,244,161	34
Unknown		6.085	333,273	-
Flavonoid	Flavonol derivatives	7.008	315,269	35
Fatty acid	Decanoic acid	8.325	171,127	-
Phenolic acid	Caffeic acid	13.980	179,161,135,109,89	35

### Antioxidant activities

Absorbance at 550 nm of cocoa pod extract was measured after at least 10 minutes of incubation at room temperature by DPPH assay to ensure complete reaction. The incubation time was determined previously (data not included) by a method as described by Marxen et al. [24]. CPE achieved optimum value of scavenging percentage by this assay at 62.5 μg/ml, which was lower than PBE and AA at 125 μg/ml (Table 3). At this optimum value, CPE showed significantly higher antioxidant activity (87.07 ± 3.09%; 62.5 μg/ml) compared with AA (72.21 ± 2.21%; 125 μg/ml) and PBE (67.43 ± 1.71%; 125 μg/ml). The effective concentration to reduce the DPPH radical to 50% (EC_50_) was determined by plotting linear regression curve of DPPH activity versus ratio of sample concentration to DPPH as previously reported [24]. The tested sample with EC_50_ value lower than 30 μg/ml by DPPH assay, has high efficiency as free radical scavenger [2], specifically against superoxide anion radicals [59], as exhibited by CPE, which was two times higher antioxidant activity than standardized PBE, although it indicated a two-fold decrease in comparison with AA. The presence of more than one carboxylic acid such as citric acid and malic acid, besides phenolic acid and other polyphenols in CPE is suggested to contribute to high antioxidant value by DPPH assay [59], compared with AA, which contained a single compound. In addition, higher antioxidant activity values by DPPH assay for CPE than PBE is also attributed to the presence of terpenoid and resveratrol compounds.

**Table 3:** **Antioxidant activities of CPE, AA and PBE based on (a) Scavenging effect using DPPH assay (b) Metal reducing ion capability, and (c) Antioxidant activity using β-carotene bleaching assay**

***Assay***	***Sample***	***Concentration, μg/ml***								***EC***_***50***_
		***7.8***	***15.6***	***31.3***	***62.5***	***125***	***250***	***500***	***1000***	
**(a) DPPH assay (%)**	AA	45.03 ± 1.74^aα^	50.14 ± 3.20^aβ^	63.05 ± 6.39^aγ^	66.63 ± 1.75^bγ^	72.21 ± 2.21^bδ^	73.45 ± 2.24^bδ^	76.48 ± 2.58^bδ^	77.53 ± 2.67^bδ^	13.25
	PBE	13.70 ± 0.27^bα^	23.92 ± 1.04^cβ^	38.49 ± 2.26^bγ^	58.89 ± 3.16^cδ^	67.43 ± 1.71^cε^	69.93 ± 1.91^bε^	73.54 ± 1.85^bε^	74.86 ± 1.42^bε^	49.33
	CPE	10.80 ± 0.63^cα^	32.10 ± 0.40^bβ^	58.98 ± 5.82^aγ^	87.07 ± 3.09^aδ^	85.01 ± 4.44^aδ^	86.81 ± 3.47^aδ^	84.52 ± 3.23^aδ^	83.28 ± 0.66^aδ^	26.10
**(b) FRAP assay (μM Fe** ^**2+**^ **)**	AA	416.24 ± 78.22^abαβ^	610.43 ± 3.99^aγδ^	405.47 ± 42.79^bαβ^	454.19 ± 31.55^cβ^	336.24 ± 45.33^cα^	534.53 ± 29.57^bγ^	599.83 ± 100.81^bγ^	754.02 ± 72.49^aγε^	204.25
	PBE	398.11 ± 15.95^bα^	485.06 ± 39.38^bβγ^	483.67 ± 53.55^aβγ^	509.22 ± 46.27^bγ^	551.44 ± 45.71^aγδ^	435.61 ± 57.16^cβγ^	793.94 ± 41.84^aε^	587.83 ± 4.71^bδ^	70.76
	CPE	478.12 ± 46.06^aβ^	567.86 ± 38.07^aγ^	390.94 ± 15.23^bα^	684.27 ± 18.72^aδε^	526.66 ± 42.06^aβγ^	620.68 ± 9.07^aδ^	638.80 ± 52.31^bδ^	729.57 ± 57.20^aε^	97.30
**(c) BCB assay (%)**	BHT	31.01 ± 3.60^aα^	46.83 ± 3.18^aβ^	59.26 ± 1.46^aγ^	78.70 ± 3.16^aδ^	91.72 ± 2.51^aε^	98.23 ± 6.09^aε^	97.17 ± 2.82^aε^	99.17 ± 5.48^aε^	24.09
	PBE	3.67 ± 0.27^cα^	12.88 ± 2.26^bβ^	23.20 ± 7.65^bγ^	25.31 ± 6.89^cγ^	40.71 ± 8.66^cγδ^	48.32 ± 7.73^cδ^	49.59 ± 3.51^cδ^	63.68 ± 13.15^cδ^	302.71
	CPE	9.65 ± 1.99^bβ^	5.15 ± 1.10^cα^	5.74 ± 1.48^cα^	51.47 ± 5.64^bγ^	70.50 ± 0.43^bδ^	81.55 ± 1.08^bε^	83.44 ± 0.80^b£^	88.08 ± 2.73^b€^	84.67

^a b c d^Different alphabet in column means significantly different within sample for each assay.

^α β γ δ ε € £^different symbol means significantly different within concentration for each sample in row for each assay.

The ability of the tested solution to deviate the mechanism of Fenton reaction by chelating the metal ions [60], such as Fe^2+^ and Cu^2+^, which is responsible to convert the hydrogen peroxide to hydroxyl radical (ROS) on the skin [6], can be measured using FRAP assay. We conducted a kinetic study for the tested sample at 620 nm (data not published) measured after five minutes of reaction. CPE had similar antioxidant activity to AA when measured using FRAP assay except at 250 μg/ml, where the FRAP value of CPE was significantly higher than AA and PBE. Antioxidant activity of PBE was proportional to the tested concentration and showed significantly higher activity when compared with EC_50_ value of the FRAP assay. Although CPE had three times higher metal reducing ion potential than AA, its performance was 1.4 times lower than that of PBE. The presence of chlorogenic acid derivatives in CPE is likely to contribute to its higher FRAP value than that of AA since this compound is metal chelating ion [60]. Other than that, flavonol derivatives are also known to chelate metal ions [25], such as rhamnetin and kaempferol derivatives in CPE. Flavanol compounds, such as catechin and quercetin also contribute to the chelating action on metal ion [26], which are available in PBE but absent in CPE, resulting in higher FRAP value for PBE. Phenolics with ion reducing ability diminish the possibility of hydroxyl radical’s formation path from superoxide anion radicals and additionally inhibit enzymes due to their abilities to chelate copper at the active site [6].

β-carotene in BCB assay serves as an indicator that degrades during the oxidation process when linoleic acid turns to hydroperoxides at high incubation temperature [61]. The presence of antioxidant eliminates or reduces the action of this radical species to β-carotene as measured by the absorbance at 450 nm and calculation of degradation rate. High value of antioxidant activity by BCB assay exhibits the tested solution is a good antioxidant agent due to the presence of linoleic acid that acts as pro-oxidant [21]. CPE achieved optimum value of antioxidant activity at concentration of 250 μg/ml (81.55 ± 1.08%), which was higher than standardized PBE and BHT at 125 μg/ml (40.71 ± 8.66% and 91.72 ± 2.51%, respectively). Both CPE and PBE had lower antioxidant activity than BHT, but based on EC_50_ value; antioxidant activity of CPE was four times lower than that of BHT while PBE showed the lowest activity. Higher antioxidant activity of CPE in comparison with PBE can be attributed to the terpenoid and resveratrol compounds that solely indicate better protection for lipid oxidation [62, 63]. Plant extracts with lipid protection properties can protect the skin from lipid peroxyl radicals that attack stratum corneum layer [63].

### Elastase and collagenase inhibition

Determination of elastase inhibition at higher concentration (1000 μg/ml) of CPE and standardized PBE resulted in a negative slope, which was also observed for 500 μg/ml of AA, indicating no activity of elastase either for inhibition or activation. This situation should be taken into consideration to avoid false positive results, where the inhibition detects at certain concentration range of extracts or control inhibitors; i.e. elastatinal (100 μM) as in Enzo Life Science Manual [27] as well as the concentration of enzyme (neutrophil elastase enzyme, 100 μM) used in this study. Table 4 summarizes the result of elastase inhibition for CPE and standardized PBE at concentrations of 500, 100 and 10 μg/ml. At high concentrations (500 and 100 μg/ml), standardized PBE exhibited higher activity than control inhibitor, which was in agreement with a previous report by Grimm et al. [64]. Catechin, a flavanol was reported to have an inhibitory effect on elastase enzyme [65, 66], which is also present in PBE. Catechin was also reported to inhibit binding activity of transcription factors and kinases (AP1 and NF-κB), therefore lowering the activity of MMPs for collagen degradation and inflammation [67]. CPE has lower elastase inhibition activity in comparison with standardized PBE at 100 μg/ml but similar to AA and control inhibitor. Terpenoid compounds were previously suggested as elastase inhibitors [65], is present in CPE since flavones and flavonol components are not efficient inhibitors of elastase activity [66]. The inhibition of elastase by CPE was much lower at lower concentration (10 μg/ml) in comparison with AA although higher than standardized PBE. Therefore, CPE has low activity to inhibit the elastase enzyme which is responsible for degradation of elastin fibrous structure in a dermal matrix.

**Table 4:** **Elastase inhibition activity with elastatinal at 100 μM for positive control inhibitor for CPE, AA and PBE**

***Sample***	***Concentration (μg/ml)***			***IC***_***50***_
	***10***	***100***	***500***	
Elastatinal, 100 μM	30.94 ± 1.47^b^			-
AA	36.02 ± 1.83^aβ^	27.51 ± 2.59^bα^	-	232.29
PBE	8.57 ± 0.63^dα^	34.96 ± 1.47^aβ^	43.28 ± 0.05^aγ^	31.93
CPE	15.57 ± 0.86^cβ^	23.47 ± 2.15^bγ^	2.54 ± 0.04^cα^	3.51

^a b c d^Different alphabet in column means significantly different within sample.

^α β γ δ ε € £^different symbol means significantly different within concentration for each sample in row.

CPE exhibited almost two times better collagenase inhibition activity than AA and three times better activity than standardized PBE based on IC_50_ (Table 5). Increasing the concentration of the tested sample to more than 1000 μg/ml (data not shown) did not affect the collagenase inhibition activity, as exhibited by standardized PBE. Research by Lim et al. [68] indicated that kaempferol and quercetin were strong inhibitors of collagenase while apigenin, a flavone compound, weakly inhibited collagenase activity. Flavonols had better inhibition activity towards collagenase enzyme than flavones compounds [69]. Resveratrol, a stilbenoid compound, was patented for its effective inhibition against the collagenase effectively in reducing wrinkles [70]. CPE showed significantly higher collagenase inhibition activity than standardized PBE at 1000 μg/ml, although the activity was lower than that of AA and control inhibitor. The higher inhibition by CPE can be attributed to the presence of more than one flavonol compound, whereas PBE has only one identified flavonol compound. In addition, CPE was detected with two types of resveratrol compared with PBE. Therefore, the presence of various polyphenolic compounds in CPE contributed to its potential in maintaining collagen longevity in the skin layer [4].

**Table 5:** **Collagenase inhibition activity of CPE, AA and PBE with NNGH at 1.3 μM for positive control inhibitor**

Sample	***Concentration (μg/ml)***								***IC***_***50***_
	***7.8***	***15.6***	***31.3***	***62.5***	***125***	***250***	***500***	***1000***	
NNGH, 1.3 μM	95.91 ± 0.23^a^								-
AA	31.87 ± 3.40^cα^	44.06 ± 0.07^cβ^	48.70 ± 2.92^cγ^	33.84 ± 1.00^dα^	31.70 ± 2.36^cα^	61.75 ± 8.51^bcδ^	52.91 ± 4.24^aγ^	90.16 ± 1.27^aε^	261.38
PBE	54.96 ± 6.77^bδε€^	50.81 ± 0.90^bδ^	53.32 ± 0.53^bε^	54.04 ± 1.48^bε^	16.79 ± 2.27^dα^	60.56 ± 4.98^c€^	46.00 ± 1.64^bγ^	39.26 ± 3.98^cβ^	356.01
CPE	53.15 ± 0.95^bδ^	48.81 ± 2.55^bγ^	53.23 ± 0.41^bδ^	50.71 ± 0.31^cγ^	38.53 ± 4.22^bβ^	68.71 ± 2.12^bε^	29.97 ± 1.36^cα^	71.44 ± 2.28^bε^	111.29

^a b c d^Different alphabet in column means significantly different within sample.

^α β γ δ ε € £^different symbol means significantly different within concentration for each sample in row.

### Skin whitening and UV-protecting potential

The capability of CPE to inhibit tyrosinase activity can be translated to its potential as skin whitening agent. When tyrosinase enzyme activity is inhibited, melanin production is reduced, resulting in a fairer skin. Inhibition of tyrosinase activity was significantly higher in CPE than standardized PBE at high concentration (1000 μg/ml) but showed similar activity at lower concentration (125 μg/ml). CPE was also found to be a better inhibitor of tyrosinase activity than kojic acid and ascorbic acid (Table 6), although not as good as PBE based on the IC_50_ value. CPE and PBE contained several compounds, such as flavonols, flavanols, stilbenoids and phenolic acid, reported to inhibit tyrosinase activity [6]. Quercetin, a flavonol compound, inhibits tyrosinase enzyme better than kaempferol [71] suggesting higher inhibition activity by PBE [58] than CPE. Resveratrol in CPE contributed to the inhibition of the enzyme; however, this contribution was less than that of oxyresveratrol [72]. Fatty acids, such as linoleic acid and oleic acid, which were detected in the CPE too, were also reported to have skin whitening properties without adverse effects [73].

**Table 6:** **Skin whitening activity (%) of CPE, AA and PBE using mushroom tyrosinase against kojic acid (KA)**

***Sample***	***Concentration, μg/ml***								***IC***_***50***_
	***7.8***	***15.6***	***31.3***	***62.5***	***125***	***250***	***500***	***1000***	
**KA**	18.07 ± 0.25^bβ^	22.44 ± 0.04^bγ^	11.17 ± 1.71^cα^	11.71 ± 1.27^cα^	24.12 ± 4.89^bγ^	52.22 ± 7.46^bδ^	47.78 ± 4.07^bδ^	68.80 ± 2.89^cε^	572.28
**AA**	8.29 ± 0.81^cβ^	7.25 ± 0.08^cα^	14.15 ± 2.88^bγδε^	13.51 ± 1.40^c δ^	11.84 ± 0.47^cγ^	25.26 ± 0.89^d^	18.61 ± 2.71^cε^	29.62 ± 1.51^d€^	670.82
**PBE**	42.57 ± 1.98^aβγ^	41.22 ± 2.21^aβ^	43.14 ± 2.78^aβγ^	44.16 ± 0.79^aγ^	48.31 ± 2.71^aδ^	36.67 ± 0.40^c α^	49.10 ± 2.97^bδ^	77.65 ± 4.51^bε^	315.16
**CPE**	16.17 ± 2.67^bα^	21.52 ± 2.29^bβ^	16.97 ± 3.48^bαβ^	27.78 ± 3.19^bγ^	41.99 ± 6.93^aδ^	62.23 ± 0.47^aε^	65.87 ± 4.20^aε^	86.51 ± 2.12^a €^	357.95

^a b c d^Different alphabet in column means significantly different within concentration.

^α β γ δ ε € £^different symbol means significantly different within concentration for each sample in row.

Absorbance at UVC spectrum (200-290 nm) is not discussed as the radiation is filtered by the atmosphere. Meanwhile, absorbance of the tested samples at the other two spectrums; i.e., UVB (290-315 nm) and UVA (315-400 nm), are discussed. There are two ways in which sunscreen agent in cosmetic products protect the skin: (i) by scattering the UV-rays (titanium and zinc oxide) or (ii) by absorbing the light before reaching the skin. Plant extract, especially with flavones [74] and pigments, can absorb UV light [67]. CPE showed potentially good UV absorbance at UVB range wavelength, which was even better than the commercially used UV-protecting agent in this study; i.e. Avobenzone and Octylmethoxycinnamate (OMC), and significantly better than standardized PBE at 290 nm (Table 7). Higher sun-protecting action in CPE could be caused by flavone derivatives such as luteolin [75] at UVA wavelength (330 nm), which were absent in PBE. The presence of methyl salicylate compound in CPE also enhanced the absorbance [76]. CPE also showed similar absorbance potential to that of commercial UV-sunscreen agent, OMC, at 308 nm, the peak of erythema (sunburn) action spectrum [77]. The extract had significantly better absorbance than Avobenzone at this wavelength.

**Table 7:** **Absorbance of CPE at critical wavelength in comparison with commercial sunscreen agents and standardized pine bark extract**

***Sample***	***Wavelength (nm)***			
	290 (UVB)	308 (UVB)	330 (UVA)	350 (UVA)
***Avobenzone***	2.080 ± 0.033^c^	1.926 ± 0.052^b^	2.519 ± 0.091^a^	2.723 ± 0.247^a^
***OMC***	2.145 ± 0.002^b^	2.126 ± 0.030^a^	2.413 ± 0.124^a^	2.796 ± 0.145^a^
***PBE***	0.589 ± 0.009^d^	0.231 ± 0.005^c^	0.126 ± 0.003^c^	0.063 ± 0.002^c^
***CPE***	2.639 ± 0.010^a^	2.130 ± 0.120^a^	1.566 ± 0.200^b^	1.007 ± 0.150^b^

^a b c d^Different alphabet in column means significantly different within sample at each wavelength.

**Figure 1:**
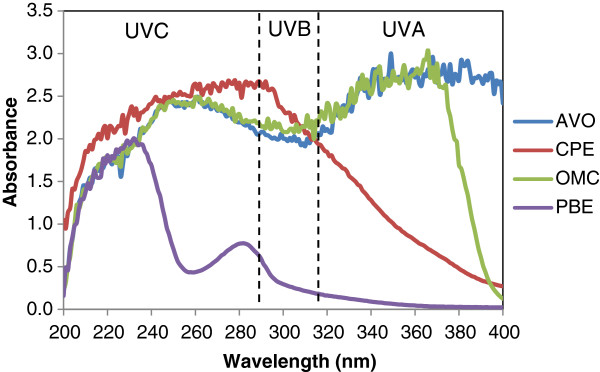
**Absorbance of tested samples compared with commercial UV-sunscreen agent (Avobenzone and Octylmethoxycinnamate) at 200–400 wavelength.**

CPE had moderate absorbance of UVA (315-400 nm) as illustrated in Figure 1, which was significantly better than that of standardized PBE (Table 7). Therefore, CPE has better potential as UVB sunscreen agent for cosmetic formulation and can be recommended in combination with other sunscreen agents with higher UVA absorbance properties. All plant extracts with pigment and color can absorb UVA and or UVB [67] as CPE does. Extracts with color might not be possible for white cream formulation; however, for color cosmetic products; i.e. foundation and lipstick, colored extracts could be added as sunscreen agents.

### Cell viability using human dermal fibroblasts

In this study we calculated the IC_90_, the concentration of the tested solution to maintain the cell population up to 90%. High value of IC_90_ indicated that high concentration of the tested solution is needed to maintain the population of fibroblast cell. Table 8 shows that CPE had the lowest value indicating low concentration of the extract was able to maintain the cell growth at 90%. The highest value obtained by PBE, which was almost two times more than that of AA. Almost no toxicity was exhibited by CPE at the tested concentration since the cell viability percentage was more than 50%, in comparison with AA and PBE. There could be possible cell death at low concentrations for AA at 62.5 μg/ml and PBE at 31.3 μg/ml, in contrast to the results of a study conducted by Kim et al. [58] in which PBE showed no toxicity to B16 melanoma cell, a type of cell different from the one used in this study. At high concentrations of AA and PBE, the cells proliferated as shown by the increasing percentage of more than 100%, the highest percentage being achieved at 500 μg/ml. Contrary to CPE, at low concentration of the tested solution (3.9 μg/ml), the percentage of cell viability was the highest, thus low concentration of CPE was needed to induce proliferation of fibroblast cell.

**Table 8:** **Cell viability (%) of the CPE against HDFa in comparison with PBE and AA**

***Sample***	***Concentration, μg/ml***									***IC***_***90***_
	***3.9***	***7.8***	***15.6***	***31.3***	***62.5***	***125***	***250***	***500***	***1000***	
**AA**	14.35 ± 3.04^bα^	18.70 ± 1.84^c α^	22.17 ± 0.61^c β^	36.96 ± 4.30^aγ^	40.87 ± 3.69^cδ^	108.26 ± 24.90^aε^	590.43 ± 19.68^a€^	1407.39 ± 46.12^a£^	651.30 ± 133.04^a€^	57.10
**PBE**	17.95 ± 1.21^bα^	24.79 ± 2.42^bβ^	29.49 ± 1.81^bγ^	42.31 ± 5.44^aδ^	69.23 ± 1.21^aε^	118.35 ± 1.81^a€^	202.14 ± 42.91^b£^	335.47 ± 17.53^bζ^	285.47 ± 49.56^b£ζ^	101.21
**CPE**	368.47 ± 46.15^aγ^	62.32 ± 4.49^aβ^	40.80 ± 7.41^aα^	39.92 ± 2.74^aα^	55.62 ± 8.32^bβ^	58.47 ± 6.26^bβ^	61.75 ± 11.20^cβ^	76.83 ± 12.25^cβ^	62.82 ± 10.11^cβ^	7.42

^a b c d^Different alphabet in column means significantly different within concentration.

^α β γ δ ε € £^different symbol means significantly different within concentration for each sample in row.

## Conclusions

We concluded that cocoa pod extract exhibits better antioxidant activities potential than standardized pine bark extract as measured using DPPH scavenging and β-carotene bleaching assays, but lower activities using FRAP assay, based on EC_50_. CPE inhibited collagenase and elastase enzymes better than PBE, although second to PBE in inhibiting the tyrosinase enzyme. Cocoa pod extract also possesses high potential as UVB sunscreen agent although with lower performance in UVA range wavelength than that of the commercial sunscreen agent. However, the extract can be formulated with other UVA protecting agents to work synergistically for a broad spectrum of UV exposure. We, therefore, recommend a possible use of CPE as ingredient for functional cosmetic products specifically for anti-wrinkles as well as skin whitening or sunscreen products in combination with natural plant extracts to widen the spectrum of protecting from sun-rays.

## Abbreviations

AA: Ascorbic acid
BCB: β-carotene bleaching
BHT: Butylated hydroxytoluene
CPE: Cocoa pod extract
DMEM: Dulbecco’s modified Eagle’s medium
DMSO: Dimethyl sulfoxide
DNTB: Dinitrothiocyano benzene
DPPH: 2-diphenyl-2-picrylhydrazyl
EC_50_: Effective concentration at 50%
ECM: Extracellular matrix
FBS: Fetal bovine serum
FRAP: Ferric Reducing Antioxidant Power
GAE: Gallic Acid Equivalent
GS1: Nebulising gas
GS2: Heating gas
HDFa: Human dermal fibroblast adult
HEPES: Hydroxyethyl piperazineethanesulfonic acid
IC_50_: Inhibition concentration at 50%
IC_90_: Inhibition concentration at 90%
KA: Kojic acid
LC-MS/MS: Liquid chromatography mass spectrometry/mass spectrometry
L-DOPA: L-3,4-dihydroxyphenyl-alanine methyl ester hydrochloride
MMP-1: Matrix metalloproteinase-1
MS IDA EPI: Mass spectrometry information dependant acquisition enhanced product ion
MTT: 3-(4,5-Dimethylthiazol-2-yl)-2,5-diphenyl tetrazolium bromide
NNGH: N-Isobutyl-N-(4-methoxyphenylsulfonyl) glycylhydroxamic acid
OMC: Octylmethoxycinnamate
PBE: Pine bark extract
PBS: Phosphate buffer saline
ROS: Reactive oxygen species
TFC: Total flavonoid content
TPC: Total phenolic content
TPTZ: 2,4,6-tripyridyl-s-triazine
UHPLC: Ultra high performance liquid chromatography
UV: Ultraviolet.
